# Carbon storage shifts around Antarctica

**DOI:** 10.1038/s41467-022-31152-3

**Published:** 2022-06-14

**Authors:** Michael P. Meredith

**Affiliations:** British Antarctic Survey, Cambridge, UK

**Keywords:** Ocean sciences, Climate sciences

## Abstract

Dense water production in the seas around Antarctica is a key process for century-scale carbon storage, slowing global warming. Results from an advanced new model reveal the prospect of system changes that may greatly reduce the efficiency of this carbon storage by the end of this century.

As our planet continues to overheat, understanding how the ocean absorbs and stores carbon takes on ever-increasing significance. At present, global warming is attenuated by the reduction of greenhouse gases in the atmosphere as CO_2_ is drawn down and stored in the ocean. The Southern Ocean around Antarctica is disproportionately important to this, due partly to its unique pattern of circulation that facilitates the efficient transfer of waters from the surface to depth. Regions such as the Weddell Sea are key to this carbon sequestration. Here, dense waters sink from the shelf and spread to flood the global abyss, isolating transported carbon from the atmosphere at depths of several kilometers for centuries to millennia.

Writing in *Nature Communications*, Nissen et al.^1^ present results from simulations of the Weddell Sea that include ice shelf cavities and that resolve both physical and biological processes involved in deep-ocean carbon accumulation. When run under the high-emission SSP5-8.5 climate change scenario, changes to the rate and mechanism of dense water production emerge, with the potential to trigger abrupt shifts in the transfer of carbon to the greatest depths. If realised, this could impact the future partitioning of carbon between the atmosphere and ocean, potentially shortening the timescale upon which sequestered carbon can return to the surface and exacerbating climate change.

The ocean is crucial to the future climate of our planet. It has absorbed more than 90% of the excess heat now present in the climate system, and between 20-30% of total anthropogenic CO_2_ emissions since the 1980s^2^, thus buffering other parts of the Earth System from the worst impacts of global warming. A key factor in delivering this “climate favour” is the overturning circulation of the ocean (Fig. 1), whereby waters are exchanged between the surface and depths in different locations. The Southern Ocean has a critical role here. It is central to global ocean circulation and is the predominant place where waters last in contact with the atmosphere prior to the rapid increase in anthropogenic CO_2_ now return to the surface. These old waters are reprocessed into new waters, which exchange carbon with the atmosphere before sinking to depth^3^. The densest water created - Antarctic Bottom Water – spreads out to flood the global abyss. Its formation and spreading represents an efficient mechanism for carbon sequestration on time scales of centuries or longer.

**Fig. 1:**
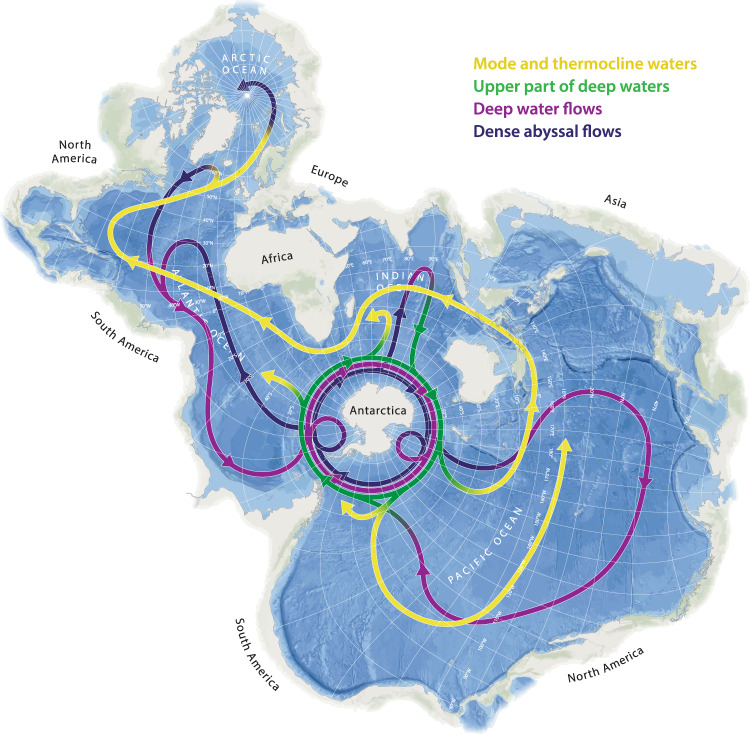
Schematic of global ocean circulation. The centrality of the Southern Ocean to the planetary-scale circulation is clear when viewed on a Spilhaus projection. It is the most prominent site globally where deep waters (purple) rise to the surface, exchange heat and carbon with the atmosphere, and then sink back to the ocean interior. The densest waters formed (black) can store carbon in the abyss for centuries or longer. This contrasts with injection into shallower layers (yellow), from which the carbon can be returned to the surface on much shorter timescales.

The stability of the Southern Ocean’s overturning circulation will strongly influence future climate change. However, investigations of this have been hampered by a historical sparsity of direct ocean measurements, especially in winter when many critical processes occur. Further, there has been great difficulty in accurately representing key elements of this circulation in models, due to the small spatial scales of important processes, and the need to resolve shelf-sea processes, including those beneath the floating parts of the Antarctic Ice Sheet.

To progress this issue, Nissen et al. develop and use an innovative model of the Weddell Sea, the most significant site of Antarctic Bottom Water formation and a region where substantial changes in carbon cycling have occurred in the recent past^4^. The model includes ice shelf cavities and an ocean carbon cycle, and, importantly, is able to form Antarctic Bottom Water more realistically than many of the models typically used to project future climate change^5^.

The model of Nissen et al. is driven by atmospheric forcing derived from a coupled climate model run under various future scenarios: a high greenhouse-gas emission future (SSP5-8.5), a constant-climate simulation, and a third simulation with constant climate but varying atmospheric CO_2_. By doing this, the impact of climatic change on the production of dense water and associated carbon drawdown is assessed. It is found that, under the high emission scenario, there is a marked reduction in deep ocean carbon transfer in the southern Weddell Sea towards the end of this century. This is driven by changes in dense water production on the southern continental shelf of the Weddell Sea, caused by a stronger inflow of warm waters from the open ocean, reduced sea ice production and freshening from stronger glacial ice melt. It leads to reduced connection between the upper and deep ocean, and hence diminished transfer of carbon to the abyss in newly-produced waters. A key factor is that, when such waters sink to shallower depths instead of the abyss, the carbon they carry can be sequestered in the ocean for shorter timescales, impacting the long-term future trajectory of climate change.

Of particular note is that, when driven under the high-emissions scenario, the model demonstrates an ability to generate some unexpectedly rapid shifts in the rate of deep-ocean carbon accumulation. These appear as an increase in the accumulation rate in the 2080s, followed by an abrupt decrease in the 2090s. These changes are traced to an intermittent switch in the mechanism by which the model produces dense waters, with the declining production on the Antarctic shelves being temporarily overcompensated by open-ocean convection over the deep Weddell Sea. Such open-ocean convection—the sinking and mixing down of dense water directly from the surface to great depth away from the continental shelf—is rare in the observational record. A pronounced example occurred in the 1970s^6^, but it was then largely absent until recent reports suggested a tentative resumption^7^. The intermittency and future intensity of dense water production by this mechanism is not well understood, and the simulations presented by Nissen et al. highlight that generating improved understanding and predictive capability is important, with added significance deriving from its impact on deep-ocean carbon storage.

The simulations by Nissen et al. have generated new insight into the potential role of changing dense water production around Antarctica in future climate change. They highlight that prospective changes in the dominant mechanisms could drive rapid shifts in the accumulation rate of deep-ocean carbon. Whilst such changes occur in their model at the end of this century, superposed on more gradual decoupling of the shelf and deep ocean environments, it is far from certain at what point the dominant modes of deep-ocean ventilation may switch in reality, or whether a threshold exists beyond which a new mode will dominate. Several recent changes around Antarctica, such as the dramatic retreat of sea ice^8^, illustrate clearly that this region is one where sudden system shifts are possible, with long-term consequences. It should be noted that Nissen et al. use a very high emission scenario (SSP5-8.5). A lower greenhouse-gas future is looking achievable, especially given recent commitments and pledges to reduce carbon emissions^9^, but action is required to translate commitments into action. Further work is needed to ascertain the likelihood of shifts in deep-ocean ventilation under more likely emissions scenarios. The possible emergence of such shifts does, however, point to potentially more variable and less predictable deep-ocean carbon sequestration in future, strengthening the imperative for further modelling and sustained observational programs in this most challenging of environments.
